# Paraphyly of the Subgenus *Sintonius* (Diptera, Psychodidae, *Sergentomyia*): Status of the Malagasy Species. Creation of a New Subgenus and Description of a New Species

**DOI:** 10.1371/journal.pone.0098065

**Published:** 2014-06-03

**Authors:** Fano José Randrianambinintsoa, Nicole Léger, Vincent Robert, Jérôme Depaquit

**Affiliations:** 1 Département de Biologie Animale, Faculté des Sciences, Université d’Antananarivo, Madagascar; 2 Université de Reims Champagne-Ardenne, ANSES, SFR Cap Santé, EA4688– USC «transmission vectorielle et épidémiosurveillance de maladies parasitaires (VECPAR)», Reims, France; 3 MIVEGEC, UMR IRD 224-CNRS 5290-UM1-UM2, Montpellier, France; 4 Institut Pasteur de Madagascar, Antananarivo, Madagascar; Wadsworth Center, United States of America

## Abstract

During an inventory of Phlebotomine sand flies carried out in Madagascar, we have identified some specimens showing morphological characters related to the subgenus *Sintonius* of the genus *Sergentomyia*. We started a molecular study based on cytochrome b mtDNA and on D1–D2 and D8 domains of the rDNA. The sampling includes all the *Sergentomyia* species available and also *S.* (*Sergentomyia*) *schwetzi*, *S*. (*Parrotomyia*) *magna*, and the following species belonging to the subgenus *Sintonius*: *S. clydei*, *S. christophersi*, *S. affinis vorax*, *S. adleri* and *S. meilloni*. The *Sintonius* subgenus (sensu Theodor) is paraphyletic. The Malagasy specimens morphologically *Sintonius*-like are never clustered with the continental *Sintonius*. We propose a new subgenus to include them: *Trouilletomyia* subg. nov. Due to the lack of mesanepisternal setae, the species *huberti* is removed from the genus *Phlebotomus* and we propose here a new combination: *Sergentomyia huberti* comb. nov. The male of *S. huberti* is pinpointed and described for the first time. Lastly, a new species for Science is described on one female: *Sergentomyia* (*Trouilletomyia*) *boironis* n. sp.

## Introduction

The subgenus *Sintonius* of the genus *Sergentomyia* was created in 1931 [1] in an article about a questionable classification of the Phlebotomine sandflies opposed to that previously proposed [2]. Curiously, *P*. (*Euphlebotomus*) *philippinensis*, and the American species *Lutzomyia gomezi* and *Nyssomyia intermedia* were included in this group [1]. Nitzulescu’s classification has not obtained the approval of any subsequent authors [3]–[7] who have considered *Sintonius* as a subgenus of the genus *Sergentomyia* França and Parrot, 1920, including the species exhibiting a cibarial armature and annealed spermathecae (designed as “*spermathèques crénelées*” by Nitzulescu). Later, Theodor considered this subgenus as an artificial group that does not sufficiently take into account the structures of the pharynx and particularly of the male genitalia. Consequently, he provided a new definition of this group [6], [7]: scanty erect hairs on abdominal tergites, style with four spines (either all terminal or two terminal and two subterminal), hooked parameres, pointed aedeagus, segmented spermathecae, buccal cavity of varying forms, pharynx lampglass shaped with a few teeth posteriorly or with ridges only.

During the last decade, we carried out several sandfly inventories in many parts of Madagascar. They included several specimens of males and females sharing the characters defining the subgenus *Sintonius*. Taking into account the high level of endemism in Madagascar [8] especially within the phlebotomine sandflies [9]–[17] before including these specimens in the subgenus *Sintonius*, we carried out a study based on two ribosomal and one mitochondrial molecular markers. This study is not a phylogenetic analysis of the subgenus *Sintonius* due to a limited sampling, nor a phylogeny of the genus *Sergentomyia*. However it demonstrates that the *Sintonius* subgenus (*sensu* Theodor) is paraphyletic. It also permitted new insights in the Malagasy sand flies with i) the creation of a new subgenus in the genus *Sergentomyia*, ii) the identification of a new species and iii) the correction of the position of one Malagasy species wrongly placed in the genus *Phlebotomus*.

## Materials and Methods

### Ethics Statement

For insect collections, we obtained a license for collecting and transporting zoological material N° 154/10/MEF/SG/DGF/DCB.SAP/SLRSE. No endangered or protected species were collected in this study.

### Sand Fly Sampling

In total, the molecular sampling encompasses 41 specimens of *Sergentomyia* from eight countries (Table 1). It includes seven species of *Sintonius sensu* Theodor, the *Sergentomyia* known from Madagascar and some other African species. They were collected using CDC miniature light traps (John W. Hock company, Gainesville, FL), ultraviolet miniature light traps, sticky traps, or Malaise traps. The traps usually run overnight from 5 p.m. to 8 a.m. the following morning.

**Table 1:** *Sergentomyia* processed.

					Genbank accession numbers		
sample	subgenus	species	collection sites		Cytb	D1–D2	D8
**SEYCH2**	*Sintonius*	*clydei*	Seychelles	Aldabra	KC669784		
**SEYCH10**					KC669792		
**CLSN2**			Senegal	Mont Rolland	KC669759	KJ721094	
**CLSN1**					KC669758	KJ721095	
**MEIL**		*meilloni*	Namibia		KJ746895	KJ721096	KJ721129
**AFBK5**		*affinis vorax*	Burkina Faso	Ouagadougou	KJ746893	KJ721097	KJ721130
**AFBK1**					KJ746894	KJ721098	KJ721131
**XXBK3**		*adleri*	Burkina Faso	Ouagadougou	KJ746879	KJ721099	KJ721132
**CHR351**		*christophersi*	Algeria	Tamanrasset	KJ746880	KJ721100	KJ721133
**MADA 1**	*Trouilletomyia* subg. nov.	*huberti* comb. nov.	Madagascar	Bemaraha-Anjohikinakina	KJ746896		KJ721134
**MADA 98**					KJ746897	KJ721101	KJ721135
**MADA 618**					KJ746881	KJ721102	KJ721136
**MADA 612**					KJ746882	KJ721103	KJ721137
**MADA 606**					KJ746883	KJ721104	KJ721138
**MADA 605**					KJ746884	KJ721105	KJ721139
**MADA 594**				Mahajanga-Anjohikely	KJ746885	KJ721106	KJ721140
**MADA 586**					KJ746886	KJ721107	KJ721141
**MADA 579**					KJ746887	KJ721108	KJ721142
**MADA 576**					KJ746888	KJ721109	KJ721143
**MADA 34**				Namoroka	KJ746889	KJ721110	KJ721144
**MADA 33**					KJ746890	KJ721111	KJ721145
**MADA 32**					KJ746891	KJ721112	KJ721146
**MADA 30**						KJ721113	KJ721134
**MADA 146**		*boironis* n.sp.	Madagascar	Isalo	KJ746892	KJ721102	KJ721135
**MADA 22**	ungrouped *Sergentomyia*	*majungaensis*	Madagascar	Namoroka	EF522778	KJ721103	KJ721136
**FR1**	*Sergentomyia*	*minuta*	France	Luberon			
**DBSN1**		*dubia*	Senegal	Mont Rolland	KJ746900	KJ721114	KJ721147
**MADA 60**	*Rondanomyia*	*goodmani*	Madagascar	Ankarana	JQ434695	KJ721115	KJ721148
**MADA 162**				Ankiliefatra	JQ421004	KJ721116	KJ721149
**MADA 161**					KJ746898	KJ721117	KJ721150
**COMO 6**		*goodmani comorensis*	Comoros	Grande Comore	JQ421017	KJ721118	KJ721151
**COMO 16**					JQ421012	KJ721119	KJ721152
**MADA 17**	*Vattieromyia*	*namo*	Madagascar	Namoroka	EU143775	KJ721120	KJ721153
**MADA 15**					EU143774	KJ721121	KJ721154
**MADA 76**		*anka*	Madagascar	Ankarana	EU143784	KJ721122	KJ721155
**MADA 69**					EU143779	KJ721123	KJ721156
**MADA 66**		*sclerosiphon*	Madagascar	Ankarana	EU143778	KJ721124	KJ721157
**MADA 65**					EU143777	KJ721125	KJ721158
**COMO 11**		*pessoni*	Comoros	Grande Comore	JQ421020	KJ721126	KJ721159
**COMO 10**					JQ421019	KJ721127	KJ721160
**46CAM**	*Parrotomyia*	*magna*	Cameroon	Daoud Safari (N-W)	KJ746899	KJ721128	
**SOU3**		*magna*	Sudan				KJ721161

Moreover, several other specimens not processed for molecular biology and the *P. huberti* holotype and paratype have been examined.

### Malagasy Study Sites

Captures have been carried out in the west and the south of Madagascar, which are subject to the trade winds with significant differences in rainfall and temperature explaining differences about climates.

**Figure 1:**
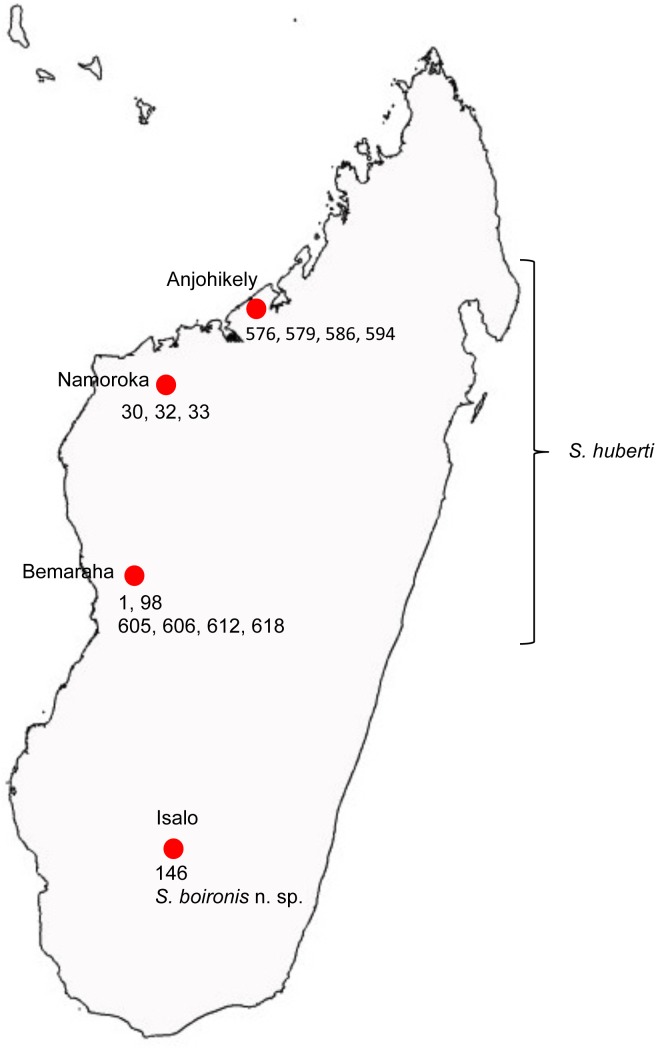
Map of Madagascar Island with the prospected localities.

The four prospected localities where sandflies have been processed in the present study are detailed below and on figure 1.

#### Anjohikely cave

This cave belongs to the Anjohibe caves complex, in the most southern part of the sandy tray Mahamavo, in the North-Western part of Madagascar at 80 km north of Mahajanga (15°33.7′S, 46°52.5′E, altitude: 100 m a.s.l.). The vegetation cover consists of “Mokoty” savannah with some patches of preserved forest on the limestone, building lapiaz. Sand flies were captured using a CDC light trap.

#### Anjohikinakina cave

It is located in the national park of Bemaraha, at 15.5 km north of Bekopaka in the western part of Madagascar (19°0′35.640″S, 44°46′3.720″E, altitude: 130 m a.s.l). The forest has dry deciduous type of limestone karst soil. The high plain of Bemaraha is a karst formation (Tsingy), located inside the reserve training pinnacles whose access is extremely difficult. Further north, can be found rolling hills interspersed with limestone rock formations. The river Manambolo limits the reserve to the south. Sand flies were captured using a CDC light trap.

#### Namoroka

This Special Reserve is located in the North-Western part of Madagascar in a dry deciduous forest on limestone karst ground. It is located within a large region from the Middle Jurassic limestone, similar to the Bemaraha’s Tsingy reserve located 250 km to the south. The limestone plateau that occupies the greater part of the reserve is in the form of a lapiaz. The south part of the Reserve is characterized by the presence of many sinkholes, some of which contain permanent pools. Sand flies have been collected using a Malaise trap near the Cave of Ambovonomby (16°28.2′S, 45°20.9′E, altitude: 200 m a.s.l).

#### Isalo

This National Park is located in the southern part of the centre of Madagascar. It is composed of Jurassic continental sandstone. The south and east of the massif consists of sandstone layers whose elements are size and resistance varies according to erosion. Sand flies were captured using a Malaise trap (22°37.60′S–45°21.49′E, altitude: 822 m a.s.l.).

### Morphological Analysis

The sand flies collected were stored in 96% ethanol. The head and genitalia were cut off in a drop of ethanol, cleared in boiling Marc-André solution and mounted between microscope slide and cover slide for species identification directly in chloral gum or after dehydration in Canada balsam. To allow long-term preservation of the specimens previously mounted in chloral gum, they were remounted in Canada balsam after complete processing of washing and dehydration. The body related to the specimen was dried and stored in a vial at −20°C before DNA extraction. The specimens were observed under a BX50 microscope and measured using the Perfect Image software (Aries Company, Chatillon, France) and a video camera connected to the microscope. Drawings were made using the *camera lucida*.

### Molecular Analysis

Genomic DNA was extracted from the thorax, wings, legs and abdomen of individual sand flies using the QIAmp DNA Mini Kit (Qiagen, Germany) following the manufacturer’s instructions, modified by crushing the sand fly tissues with a piston pellet (Treff, Switzerland), and using an elution volume of 50 to 200 µl [9].

All the mtDNA and rDNA amplifications were performed in a 50 µl volume using 5 µl of extracted DNA solution and 50 pmol of each of the primers. The PCR mix contained (final concentrations) 10 mM Tris HCl (pH 8.3), 1.5 mM MgCl_2_, 50 mM KCl, 0.01% Triton X 100, 200 µM dNTP each base, and 1.25 units of 5 prime Taq polymerase (Eppendorf, Germany). The cycle begins with an initial denaturation step at 94°C for 3 min and finishes with a final extension at 68°C for 10 min. PCRs were done with the following temperature profiles.

A fragment of cytochrome b (Cyt b): 5 cycles with 30 sec 94°C, 40 sec 40°C, 1 min 68°C and 35 cycles with 30 sec 94°C, 30 sec 44°C, 1 min 68°C using the primers N1N-PDR: 5′-CAYATTCAACCWGAATGATA-3′ and C3B-PDR: 5′-GGTAYWTTGCCTCGAWTTCGWTATGA-3′ [18];
The D8 segment of the 28S rDNA: 40 cycles with 30 sec 94°C, 40 sec 48°C, 1 min 30 sec 68°C using the primers couple C’7: 5′-GTGCAGATCTTGGTGGTAG-3′ and D8E: 5′-GCTTTGTTTTAATTAAACAGT-3′;
The D1 and D2 fragments of the 28S rDNA: 30 cycles with 1 min 94°C, 1 min 58°C, 1 min 68°C using the primers couple C1’: 5′-ACCCGCTGAATTTAAGCAT-3′ and D2: 5′-TCCGTGTTTCAAGACGGG-3′ [19].

Amplicons were analyzed by electrophoresis in 1.5% agarose gel containing ethidium bromide. Direct sequencing in both directions was performed using the primers used for DNA amplification. The correction of sequences was done using Pregap and Gap softwares included in the Staden Package [20].

We used three different data sets for phylogenetic analyses: the rDNAs D1–D2 and D8, and the mtDNA cytochrome b.

Consensus sequences were aligned by the Clustal W algorithm [21] from the BioEdit 4.8.10 sequence editor [22], and corrected manually.

### Sequences Analysis

In the present study, the sequences have been analysed using Neighbor-Joining (NJ), maximum parsimony (MP) and maximum likelihood (ML) methods. *Phlebotomus papatasi* has been used as outgroup [19], [23]–[25].

#### Neighbor-Joining (NJ)

The NJ method [26] has been analysed by MEGA software version 5 [27]. Genetic distances were corrected according to the transition/transversion rate (Kimura’s two-parameter method). Bootstrap confidence values were calculated from 1,000 replications.

#### Maximum parsimony

Maximum parsimony (MP) analysis were performed using the branch and bound option of MEGA when possible or the heuristic search. The node support was assessed by bootstrapping over 100 replications.

#### Maximum likelihood

Sequence data were analysed by PhyML [28] based on maximum likelihood. The ML trees were constructed using the substitution models selected by MODELTEST program [29] with AIC: HKY85 [30] for D1–D2 and D8, and General Time Reversible [31] for cyt b.

### Nomenclatural Acts

The electronic edition of this article conforms to the requirements of the amended International Code of Zoological Nomenclature, and hence the new names contained herein are available under that Code from the electronic edition of this article. This published work and the nomenclatural acts it contains have been registered in ZooBank, the online registration system for the ICZN. The ZooBank LSIDs (Life Science Identifiers) can be resolved and the associated information viewed through any standard web browser by appending the LSID to the prefix “http://zoobank.org/”. The LSID for this publication is: urn:lsid:zoobank.org:pub:D0300E46-E93C-4CEF-99DE-77D77E5FC442. The electronic edition of this work was published in a journal with an ISSN, and has been archived and is available from the following digital repositories PubMed Central, LOCKSS.

## Results

### Molecular Analysis

The sequences analysed in the present study have been deposited in Genbank as indicated in table 1. Despite several attempts, the direct sequencing of the *S. clydei* was not successfully performed for D8 and D1–D2 from the Seychelles specimens, like the sequencing of several markers of the specimens 1, 30 and 33, due to the small quantity of DNA extracts.

**Figure 2:**
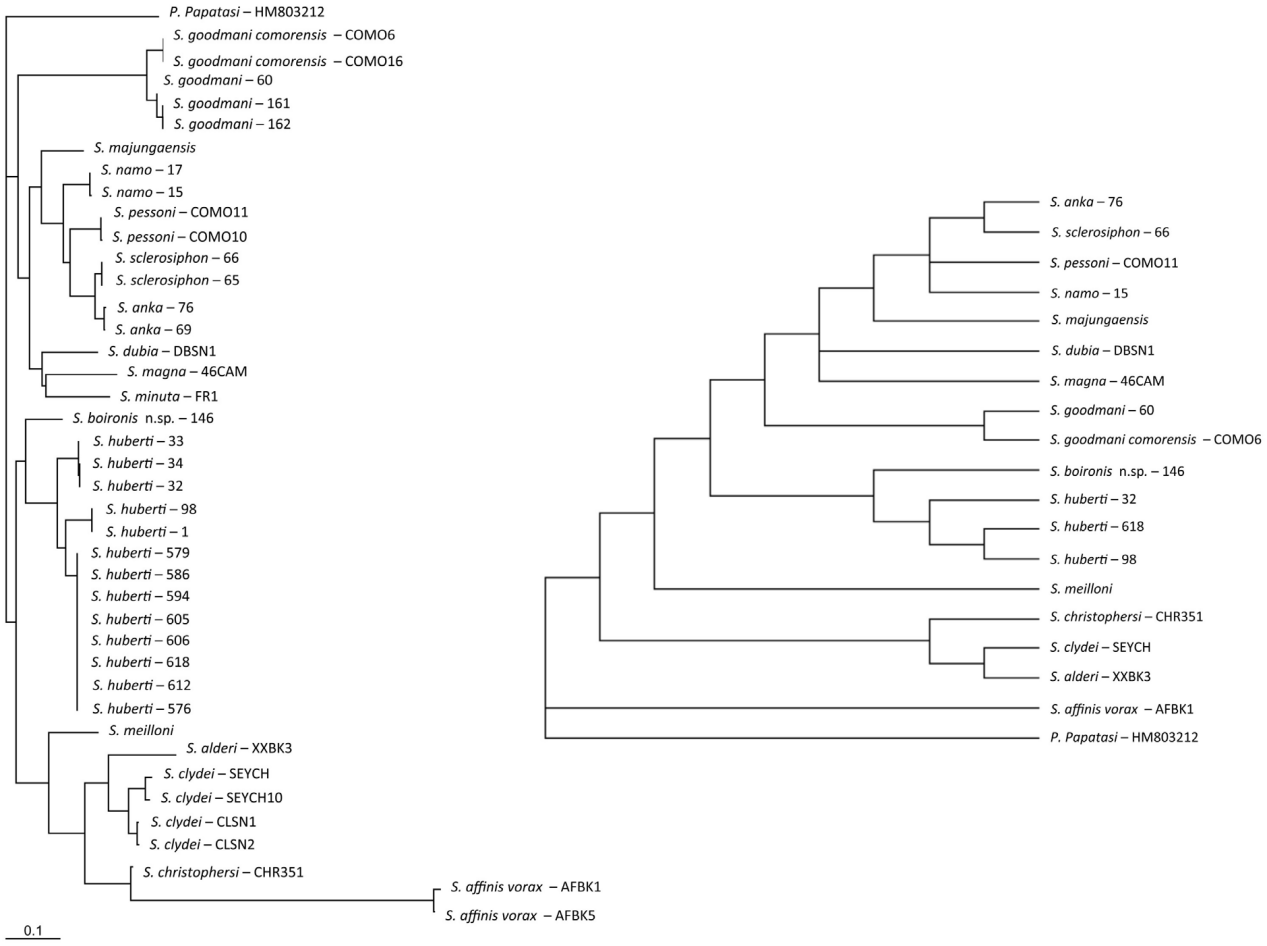
ML and MP trees based on cyt b sequences mtDNA. Bootstrap values indicated have been obtained after 100 replications.

**Figure 3:**
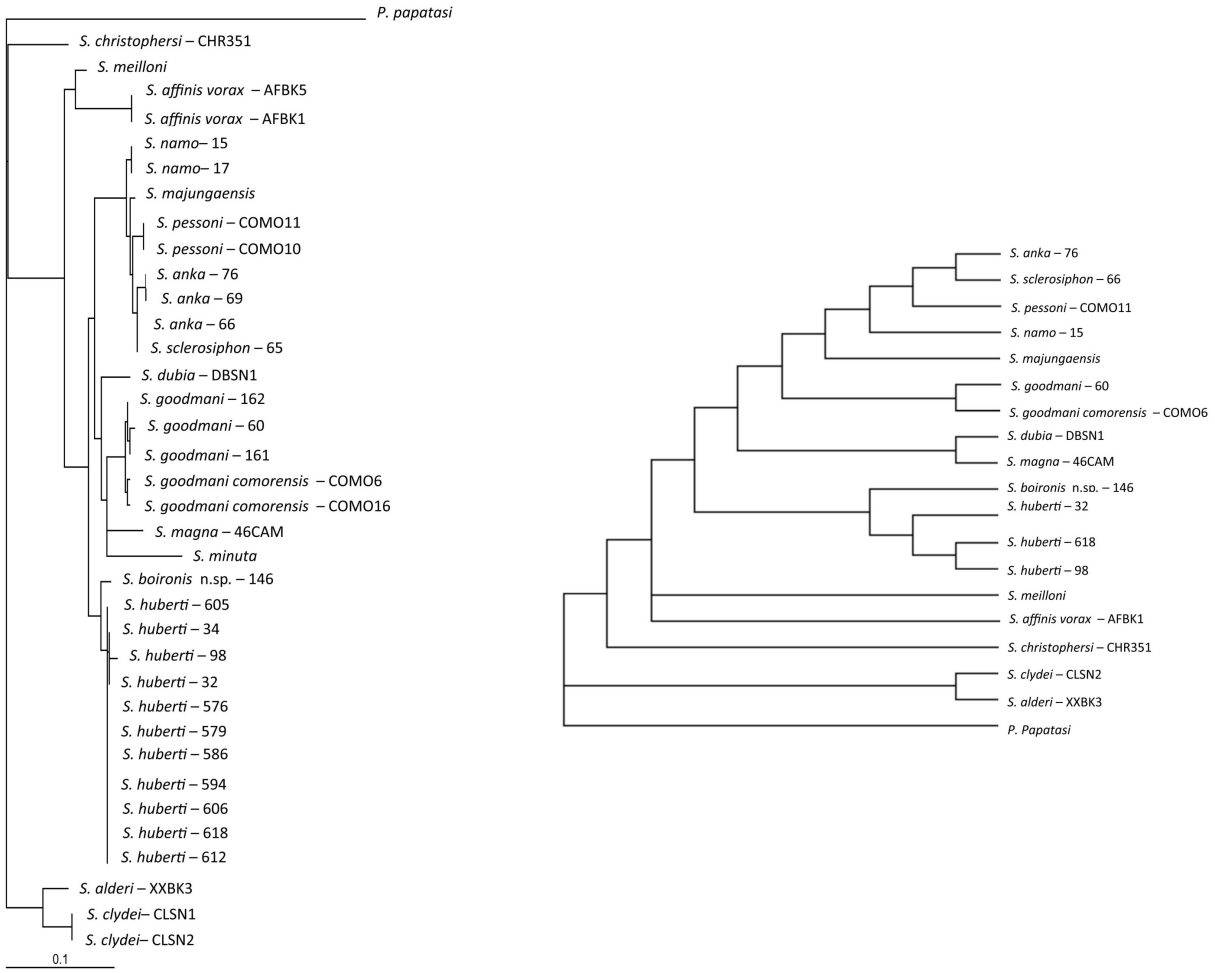
ML and MP trees based on D1–D2 sequences rDNA. Bootstrap values indicated have been obtained after 100 replications.

**Figure 4:**
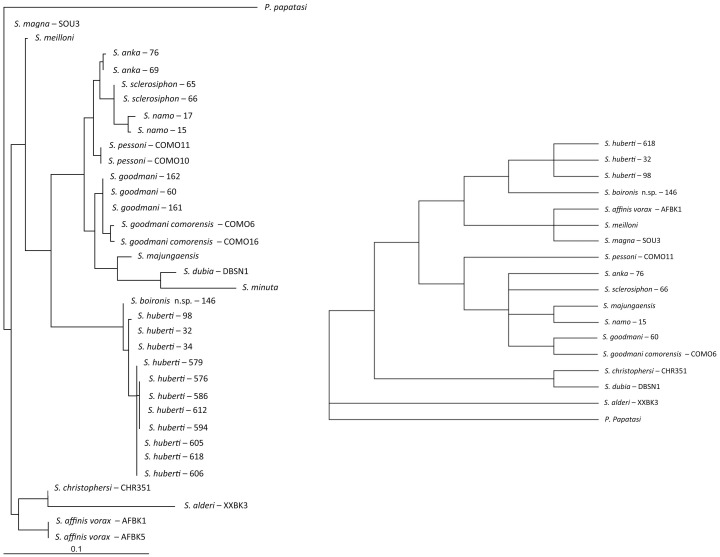
ML and MP trees based on D8 sequences rDNA. Bootstrap values indicated have been obtained after 100 replications.

A high degree of homology is observed between the topologies obtained by ML and NJ analyses. Consequently, the NJ trees are not shown. The ML and MP trees related to the analysis of the sequences of D1–D2, D8 and cyt b are shown on figures 2, 3, and 4, respectively.

All the markers and analyses isolate the Malagasy specimens exhibiting *Sintonius* morphological specimens from Anjohikinakina cave-Bemaraha (specimens 1, 98, 605, 606, 612, 618), from Anjohikely cave (specimens 576, 579, 586, 594), from Namoroka (specimens 30, 32, 33, 34) and from Isalo (specimen 146) in a clade not linked with the African *Sintonius*.

The females from Anjohikinakina cave and Bemaraha (holotype and topotypes) are morphologically similar to those from Anjohikely cave and from Namoroka. Sequences of males and females from these localities show 100% homology regarding D8 marker, a few differences for D1–D2 and cyt b sequences. Regarding the latter, differences are observed between localities, except for the specimens caught in the Bemaraha, divided in two populations. Consequently, we consider that these specimens belong to the same species. The 100% homology between the cyt b sequences of the male specimen number 98 and the female specimen number 1 (*P. huberti* holotype) allows us to describe the male of this species (see below).

The pairwise distance between the female specimen 146 from Isalo and the *S. huberti* populations (table 2) is high and is phylogeneticaly isolated (figs 2, 3 and 4). Moreover, this specimen 146 is morphologically very different from the females from Anjohikely, Namoroka and Bemaraha. All these data support the creation of a new species (see below).

**Table 2:** Male measurements.

Bemaraha		Bemaraha			Anjohikely			Namoroka			
samples	98	605, 606, 612, 618			523, 576, 579, 582, 586, 595			32	32		
		average	min	max	average	min	max			average	
**Head**											
AIII	305.07	290.97	264.63	307.89	292.14	291.76	308.26	290.53	292.25	291.39	
AIV	138.18	132.27	119.58	142.10	133.03	123.54	142.10	137.48	136.97	137.22	
AV	145.14	138.46	124.83	151.81	140.06	130.26	151.81	145.79	141.09	143.44	
L	175.54	194.08	179.98	205.43	188.76	185.69	209.08	192.60	192.90	192.75	
AIV+AV	283.32	270.73	244.40	293.91	273.09	253.80	293.91	283.26	278.06	280.66	
P1	36.14	44.20	39.30	49.31	42.24	35.44	51.83	34.09	36.36	35.23	
P2	104.67	108.97	100.20	115.58	107.36	106.36	119.36	101.01	107.79	104.40	
P3	143.24	162.02	144.92	170.91	155.27	142.47	170.91	128.79	136.00	132.39	
P4	261.90	241.49	219.08	258.28	245.19	224.03	258.28	196.85	229.96	213.41	
P5	361.08	400.50	367.41	450.10	394.77	411.00	450.10	257.44	389.14	323.29	
**Wings**											
Length	1831.34	1822.98	1809.71	1851.39	1828.03	1824.49	1880.78		1890.00	1890.00	
Width	408.81	464.80	443.77	525.51	478.03	470.56	525.51		404.66	404.66	
α	364.94	369.35	325.53	402.84	365.91	366.73	402.84		368.36	368.36	
β	293.91	396.00	380.76	425.81	400.85	380.76	445.54		412.35	412.35	
δ	219.61	178.95	148.51	219.73	182.40	175.38	219.73		167.93	167.93	
γ	364.02	308.22	279.69	334.97	307.63	287.56	338.31		317.55	317.55	
π	113.06	161.03	124.81	183.81	156.55	124.96	182.11		192.90	192.90	
w/γ	1.12	1.51	1.42	1.59	1.50	1.64	1.55		1.27	1.27	
**Genitalia**											
Style	100.16	116.87	113.97	119.46	112.62	101.11	117.10	96.29	103.14	99.72	
Coxite length	208.54	218.39	195.62	228.07	212.65	195.62	228.07	201.46	209.84	205.65	
Paramere	137.94	157.49	149.15	171.77	154.09	149.81	177.81	144.04	145.09	144.57	
Aedeagus length	66.70	79.31	69.25	89.46	76.18	79.01	94.46	71.20	67.41	69.31	
Surstyles	188.20	200.16	183.85	221.43	198.41	185.62	229.53	189.23	218.06	203.64	
Genital filaments	∼476.250	531.74	492.32	563.26	529.11	511.40	563.26	484.03	502.89	493.46	
Genital pump		117.65	101.27	131.07	116.66	108.04	131.07	120.09		120.09	

Measurements in µm.

### Morphological Analysis

The morphological examination of the Malagasy sandflies identified as *Phlebotomus huberti* (topotypes caught in 2012 and specimens from Namoroka) as well as the reexamination of the female holotype and the paratypes show a lack of mesanepisternal setae, despite the unexplained presence of a group of four mesanepisternal setae in the original description [10]. Consequently, the species *huberti* cannot belong to the genus *Phlebotomus*. We here propose a new combination: *Sergentomyia huberti* comb. nov. based on the existence of two synapomorphies i.e. the absence of pre-apical papilla on the fifth antennal segment and presence of an opened labial furca.

### Linking Molecular and Morphological Analysis

The existence of annealed spermathecae and of small teeth on the cibarium is in agreement with the characters of inclusion in the subgenus *Sintonius* [1], [6], [7]. However, the molecular data do not support the inclusion of *S. huberti* comb. nov. in the subgenus *Sintonius*. The specimen 146 belongs to the sister species of *S. huberti*. Consequently, we propose a new subgenus including *S. huberti* comb. nov. and *S. boironis* n. sp.: *Trouilletomyia* subg. nov.

Description of *Trouilletomyia* subg. nov., depaquit and léger

According to the monophyly of the Malagasy species *Sintonius*-like, we have created for them the *Trouilletomyia* subg. nov. in the genus *Sergentomyia*, that we define by i) annealed spermathecae, ii) an armed cibarium in both sexes, iii) a remarkable pharyngeal armature with two types of teeth, like that observed in the subgenera *Adlerius*, *Euphlebotomus* or *Anaphlebotomus pro parte* (Asiatic species) of the genus *Phlebotomus*. To our knowledge, this pharyngeal armature had never been observed in the genus *Sergentomyia*.

*Trouilletomyia* Léger & Depaquit subg. Nov. urn:lsid:zoobank.org:act:E2091673-4A89-49BD-8AAE-3C41E5137C1F

Description of S. huberti comb. nov.

Genus *Sergentomyia* Rondani et Berté, in Rondani, 1840.

Subgenus *Trouilletomyia* subg. nov.

Species *Sergentomyia huberti* comb. nov.

The description is based on the specimen number 98.

### Male (Fig. 5)

**Figure 5:**
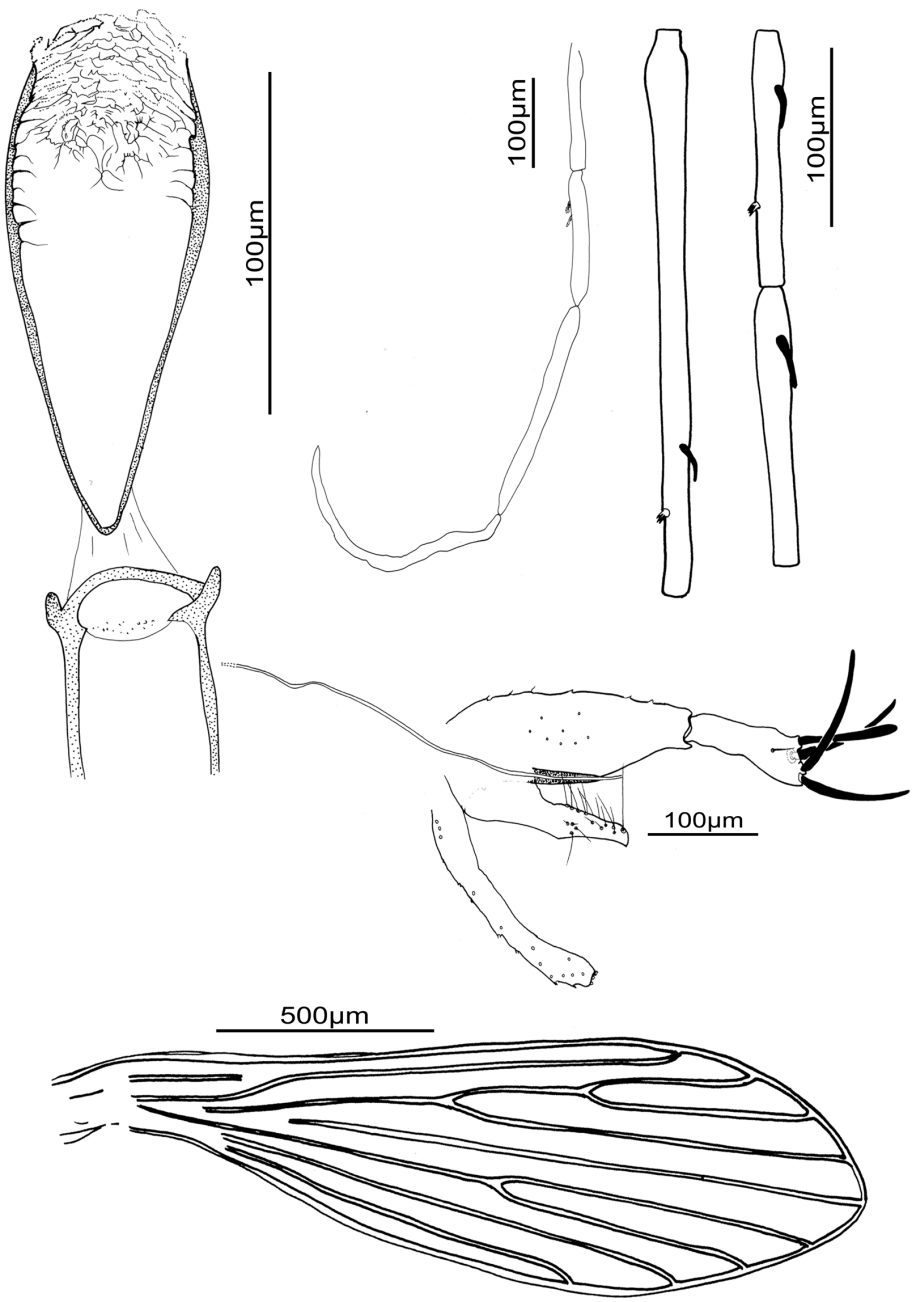
*Sergentomyia* (*Trouilletomyia*) *huberti* comb. nov. male from Bemaraha. A: Cibarium and pharynx. B: Palp. C: Antennal segments III, IV and V. D: Genitalia. E: Wing.

*Head

Inter-ocular suture: incomplete.
Cibarial armature with some discrete denticles.
Pharynx quite narrow, with a discrete armature composed of very small aligned teeth, forming ripples. Some well developed lateral teeth.
Palpal formula: 1, 2, 3, 4, 5. A few Newstead’s scales on the third palpal segment.
Antennal formula: 1/III-XV with short ascoids. AIII = 305 µm more than AIV + AV.
Labrum = 176 µm. AIII/L = 1.73.

*Thorax

No setae on the mesanepisternum.
Wing: length = 1771 µm, width = 408 µm, α = 365 µm, β293 µm = , δ = 220 µm, γ = 364 µm, π = 113 µm.
Width/γ ratio = 1.1.

*Genital Armature

Coxite 209 µm long, with about ten internal setae implanted in its middle.
Style 100 µm long, narrow, with two terminal and two subterminal spines.
Single paramere, hooked at the top.
Surstyles 188 µm long.
Aedeagus: length = 67 µm, straight, regularly tapering toward the distal end.
Genital filaments: length = 476 µm, isodiametric.

The description of female [10] remains valid, but without setae on the mesanepisternum.

Description of S. boironis n. sp. Randrianambinintsoa & Depaquit

Genus *Sergentomyia* Rondani et Berté, in Rondani, 1840.
Subgenus *Trouilletomyia* subg. nov.
Species *Sergentomyia boironis* n. sp.

***Sergentomyia boironis*** Randrianambinintsoa & Depaquit **sp. nov.** urn:lsid:zoobank.org:act: 734CFF03-B1DD-448C-92CF-1059D21D1BBC.

It is based on the specimen 146.

### Female (Fig. 6)

**Figure 6:**
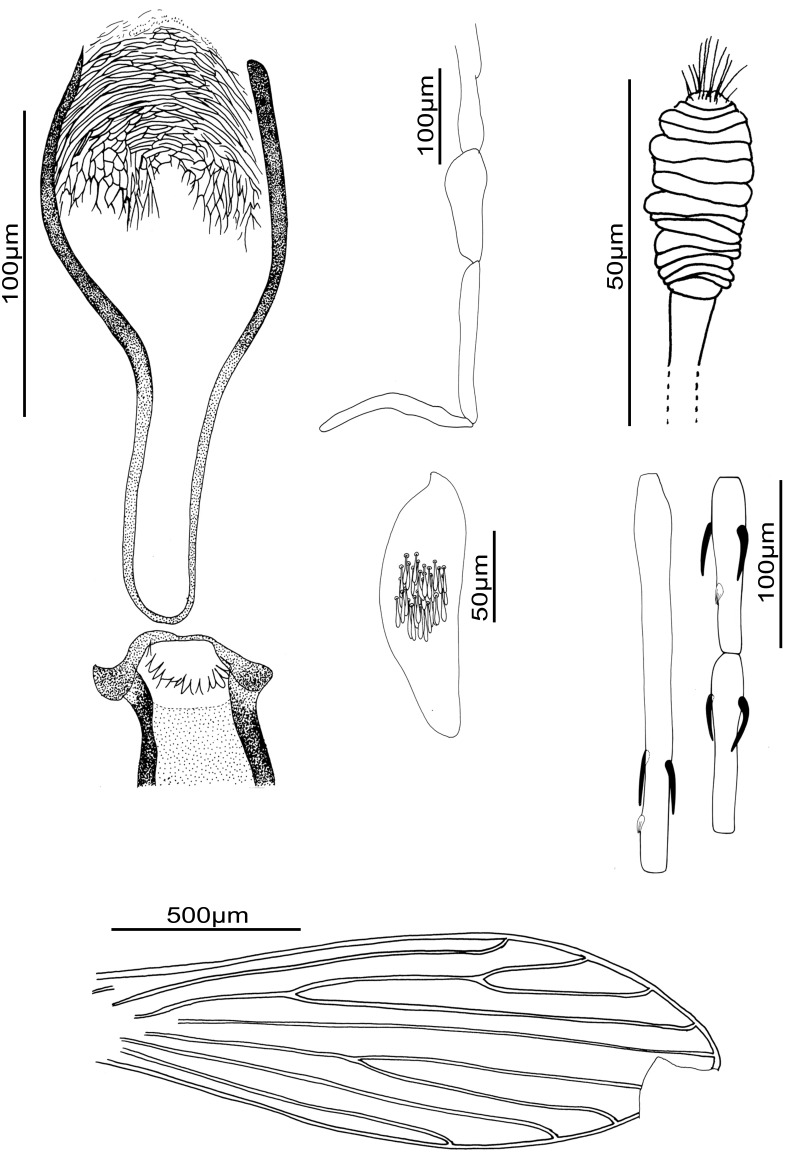
*Sergentomyia* (*Trouilletomyia*) *boironis* n. sp. female. A: Cibarium and pharynx. B: Palp. C: 3^rd^ palpal segment showing Newstead’s scales. D: Spermathecal body. E: Antennal segments III, IV and V. F: Wing.

Holotype.

*Head

Interocular suture incomplete.
Cibarial armature with fifteen pointed teeth directed backward, along an arc.
Pharyngeal armature *Adlerius* or *Euphlebotomus*-like, formed of two kinds of teeth: the posterior ones formed of several concentric ranges and the anterior ones long and oriented forward.
Palpal formula: 1, 2, 3, 4, 5. About thirty Newstead’s scales club-like in a patch on mesal face of the third segment.
Antennal formula: 2/III-XV. Short ascoids. A3 = 231 µm, longer than A4 ( = 103 µm) + A5 ( = 105 µm). Antennae lost during the remounting.
Labrum = 216 µm. A3/L = 1.07.

*Thorax

No setae on the mesanepisternum.
Wing: length about 1900 µm, width = 510 µm, β = 501 µm, δ = 79 µm, γ = 342 µm, π = 139 µm.
Width/γ ratio = 1.49.

*Spermathecae: annealed body formed by 15 rings. Rounded head, slightly invaginated in the last ring. Observation of ducts not possible.

Type-locality : Isalo National Park, Madagascar: 22°37.60′S–45°21.49′E, altitude: 822 m a.s.l.

The holotype has been deposited in the department of entomology of the Muséum National d’Histoire Naturelle, Paris.

The male remains unknown.

*Derivatio Nominum*

The subgenus *Trouilletomyia* subg. nov. is dedicated to our colleague Jean Trouillet.

The species *Sergentomyia boironis* n. sp. is dedicated to our colleague Pascal Boireau.

To meet the criteria of availability, the authors Randrianambinintsoa & Depaquit are responsible of the name *Sergentomyia boironis* n. sp. and the authors Depaquit, Léger & Randrianambinintsoa are responsible of the name *Trouilletomyia* subg. nov. and should be cited as the sole authority of these taxa, according to the Article 50(1) of the lnternational Code of Zoological Nomenclature, 4th edition, 2000.

## Discussion

Within the Phlebotominae of the Old World, the genus *Sergentomyia* França & Parrot, 1920 appears to be a catch fall group, including all the Old World species excluded from all other genera (*Phlebotomus*, *Idiophlebotomus*, *Chinius*, *Spelaeophlebotomus*, *Grassomyia*, *Parviden*s, *Spelaeomyia* and *Demeillonius*) [24], [25], [32]. Species of the genus *Sergentomyia* share the following characters: a mesanepisternum without setae, abdominal tergites 2–6 carrying usually all or most recumbent hairs, an usual 1/III–XV antennal formula in the males and 2/III–XV in the females with some exceptions, a cibarium with an armature of teeth and/or denticled more developed in females than in males (beyond exceptions), a single paramere, a style with four terminal spines (or often 2 terminal and 2 subterminal) and an accessory spine.

The genus *Sergentomyia* is regularly mentioned as probable vector of leishmaniases [33], [34] and arboviruses [35], [36], it is important that the systematics of this group is well assessed.Currently, mainly based on the spermathecal morphology, the genus *Sergentomyia* is divided in seven subgenera: *Sergentomyia* França & Parrot, 1920; *Neophlebotomus* França & Parrot, 1920; *Sintonius* Nitzulescu, 1931; *Parrotomyia* Theodor, 1948; *Rondanomyia* Theodor, 1948; *Capensomyia* Davidson, and; *Vattieromyia*, Depaquit, Léger & Robert, 2008. However many species remain unclassified at subgeneric level.

The species *S. huberti* and *S. boironis* n. sp. show the characters of inclusion in the subgenus *Sintonius* [1], [6], [7]: i) scanty erect hairs on the abdominal segments II to VI, ii) annealed spermathecae, iii) a pointed aedeagus and iv) a style with two terminal and two subterminal spines. However, the markers and molecular analyses show that *S. huberti* and *S. boironis* n. sp. never cluster with the *Sintonius* included in the present study. Moreover, the latter group is paraphyletic. Regarding the different analyses, it appears the consistency index of cyt b analysis is low (0.41) whereas those calculated from the ribosomal markers D1–D2 and D8 are higher (0.75 and 0.72), respectively. Consequently, the ribosomal markers are more reliable than cyt b. This mitochondrial marker includes many homoplasic characters and the cyt b trees could be considered as doubtful. According to D1–D2 (figure 2), the MP and ML analyses show *S. clydei* as the sister species of *S. adleri*. The relationships between *S. meilloni* and *S. affinis vorax* are not resolved by MP and the phylogenetic position of *S. christophersi* differs. Regarding the D8 rDNA domain, the positions of the *Sintonius* differ according to MP and ML trees, especially the position of *S. affinis vorax* and *S. meilloni*. The paraphyly of the subgenus *Sintonius* is proven but a more extensive study comparing morphological and molecular analyses is needed in order to revise the group. In our opinion, the main morphological traits characterising the subgenus *Sintonius* could be symplesiomorphies. For example, many species of *Phlebotomus* or American sandflies have annealed spermathecae. These data did not allow us to include *S. huberti* and *S. boironis* n.sp. in the subgenus *Sintonius*.

The specific value of *S. boironis* n. sp. is supported by both morphological and molecular data. Its cibarial armature is very different from that of *S. huberti* and we note the head of the spermathecae is rounded and included in the most distal ring, differing from that of *S. huberti* and *Sintonius*. Moreover, phylogenetical analyses show *S. boironis* n. sp. is the sister species of *S. huberti*. The pairwise distances are high between *S. boironis* n. sp and *S. huberti*: 9.5 to 12.8% for cyt b and 12 to 19% for D1–D2 (table 2). The low values (0.3 to 0.5%) observed for D8 are explained by the MEGA algorithm not taking into account the positions including indels, which support the variability between *S. boironis* n. sp. and *S. huberti*.

**Figure 7:**
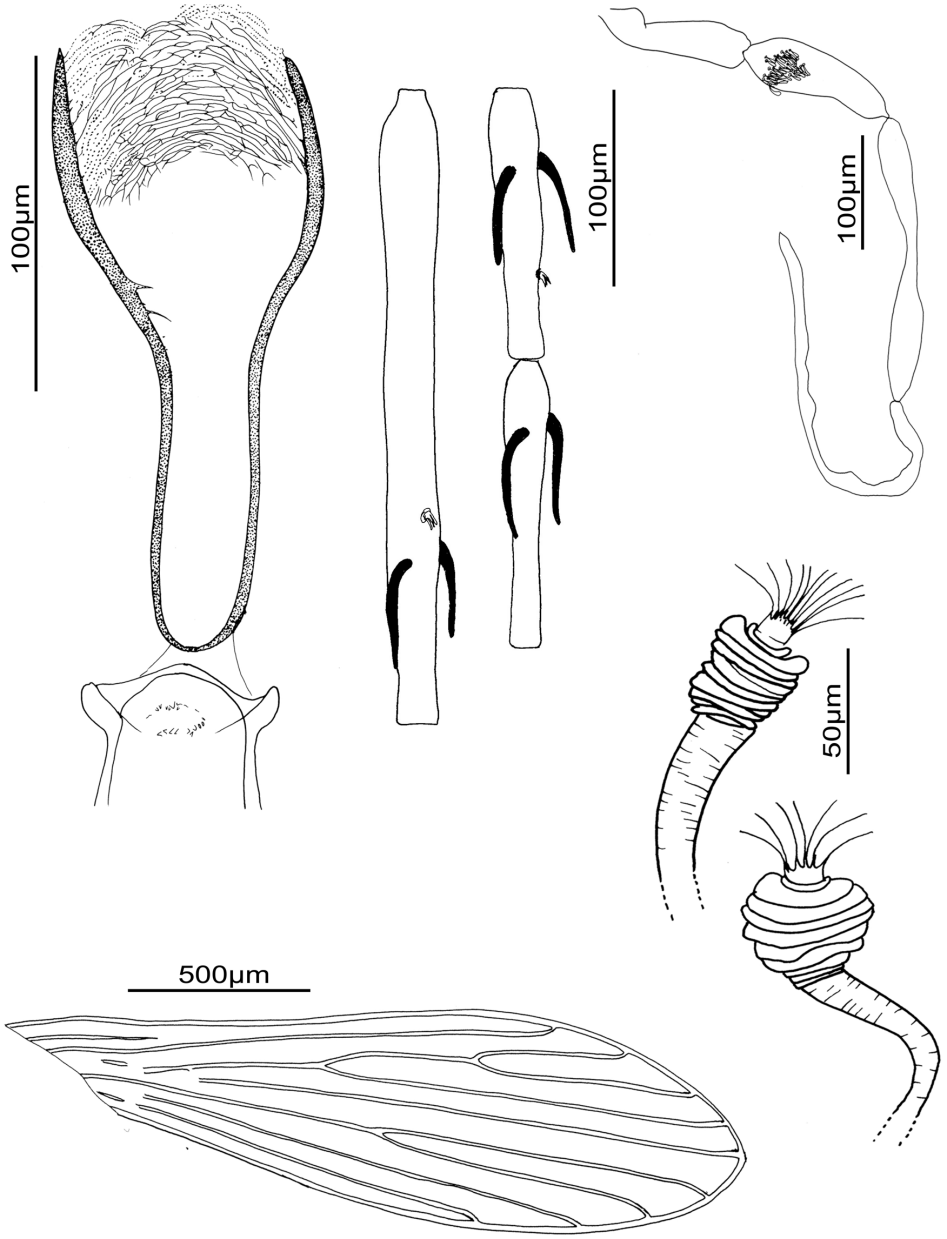
*Sergentomyia* (*Trouilletomyia*) *huberti* comb. nov. female from Namoroka. A: Cibarium and pharynx. B: Antennal segments III, IV and V. C: Palp. D. Spermathecae. E: Wing.

**Figure 8:**
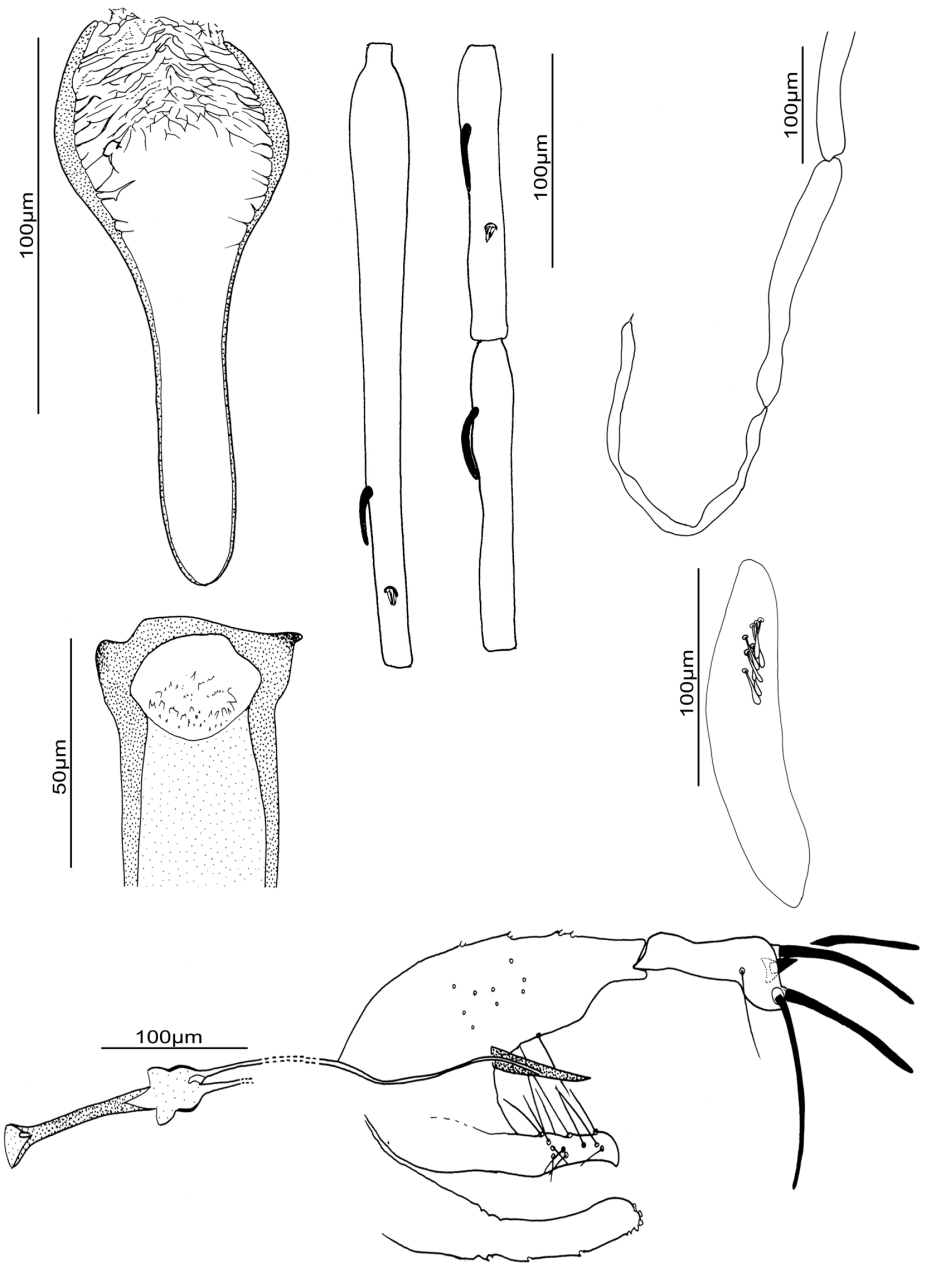
*Sergentomyia* (*Trouilletomyia*) *huberti* comb. nov. male from Namoroka. A: Cibarium. B: Pharynx. C: Antennal segments III, IV and V. D: Palp. E: 3^rd^ palpal segment showing Newstead’s scales. E: Genitalia.

We observe important molecular cyt b variability between the different populations of *S. huberti* comb. nov. However there is not enough material available from different populations and genders in order to assess if they belong to cryptic species or not. The observation of specimens from Namoroka shows little differences in females (figure 7) and males (figure 8) especially regarding the higher number of cibarial teeth. However, these observations do not seem significant enough at the present time to justify the creation of a new species. The morphometric descriptive statistics (table 3) do not emphasise significant differences between these populations. Despite the existence of two sympatric mitochondrial populations in the Anjohikinakina cave-Bemaraha (specimens 1 and 98 on the one hand, and specimens 605, 606, 612 and 618 on the other hand) will encourage new investigations related to this species.

**Table 3:** Pairwise genetic distances (%) between and within taxa.

Cyt b	(1)	(2)	(3)	(4)	(5)
Bemaraha (no type) (1)					
Anjohikely (2)	0.000				
Mada 1 et 98 (3)	0.011	0.011			
Namoroka (4)	0.012	0.012	0.013		
Isalo (5)	0.016	0.016	0.019	0.018	
**D1–D2**	**(1)**	**(2)**	**(3)**	**(4)**	**(5)**
Bemaraha (no type) (1)					
Anjohikely (2)	0.000				
Mada 98 (3)	0.007	0.007			
Namoroka (4)	0.001	0.001	0.006		
Isalo (5)	0.012	0.012	0.019	0.013	
**D8**	**(1)**	**(2)**	**(3)**	**(4)**	**(5)**
Bemaraha (no type) (1)					
Anjohikely (2)	0.001				
Mada 98 (3)	0.002	0.002			
Namoroka (4)	0.002	0.002	0.000		
Isalo (5)	0.005	0.005	0.003	0.003	

Identification key of the *Sergentomyia* and *Grassomyia* from Madagascar:

Females:

1- capsulated spermathecae 2
non capsulated spermathecae 3
2- spherical spermathecal capsule *Grassomyia*
spermathecae narrower in the median part *S.* (*Vattieromyia*)
3- smooth spermathecae *S. majungaensis*
segmented spermathecae 4
4- completely segmented spermathecae; well developed pharyngeal armature with two kinds of teeth 5
partially segmented spermathecae; discrete pharyngeal armature *S. goodmani*
5- cibarial armature of about 15 well developed pointed teeth, oriented backward, along a curved line *S. boironis* n. sp
cibarial armature with discrete teeth or denticle *S. huberti* comb. nov.

Males:

1- one ascoid on the third antennal segment (AIII) 2
absence of ascoid on the third antennal segment (AIII) 3
2- antennal formula 1/III-XII *S. goodmani*
antennal formula 1/III-XV *S. huberti*
3- AIII shorter than 200 µm *Grassomyia*
AIII longer than 200 µm 4
4- genital filaments shorter than 250 µm *S. majungaensis*
genital filaments longer than 250 µm *S*. (*Vattieromyia*).

## Supporting Information

Figure S1 **Neighbor-Joining tree based on cytochrome b sequences mtDNA.** Bootstrap values indicated have been obtained after 1,000 replicates.
(TIF)

Figure S2 **Neighbor-Joining tree based on D1-D2 sequences rDNA.** Bootstrap values indicated have been obtained after 1,000 replicates.
(TIF)

Figure S3 **Neighbor-Joining tree based on D8 sequences rDNA.** Bootstrap values indicated have been obtained after 1,000 replicates.
(TIF)
