# Correction: Paraphyly of the Subgenus *Sintonius* (Diptera, Psychodidae, *Sergentomyia*): Status of the Malagasy Species. Creation of a New Subgenus and Description of a New Species

**DOI:** 10.1371/journal.pone.0110347

**Published:** 2014-10-01

**Authors:** Jérôme Depaquit, Nicole Léger

The type-species is not designated in the published article. The type-species should appear in the Results as: “Type-species of this new subgenus: *Sergentomyia huberti* comb. nov.”

## References

[1] Randrianambinintsoa FJ, Léger N, Robert V, Depaquit J (2014) Paraphyly of the Subgenus *Sintonius* (Diptera, Psychodidae, *Sergentomyia*): Status of the Malagasy Species. Creation of a New Subgenus and Description of a New Species. PLoS ONE 9(6): e98065 doi:10.1371/journal.pone.0098065 24893009 10.1371/journal.pone.0098065 PMC4043648

