# Correction: Correction: Paraphyly of the Subgenus *Sintonius* (Diptera, Psychodidae, *Sergentomyia*): Status of the Malagasy Species. Creation of a New Subgenus and Description of a New Species

**DOI:** 10.1371/journal.pone.0117754

**Published:** 2015-02-17

**Authors:** The PLOS ONE Staff

## Notice of Republication

This correction was republished on December 18, 2014 to correct errors in authorship that were introduced during the typesetting process. Please download this correction again to view the correct version.

## Supporting Information

S1 File Republished corrected correction. (PDF)
