# Supplementary material for: Correction: Correction: Paraphyly of the Subgenus Sintonius (Diptera, Psychodidae, Sergentomyia): Status of the Malagasy Species. Creation of a New Subgenus and Description of a New Species
Source: PLoS One. 2015 Feb 17;10(2):e0117754. doi: 10.1371/journal.pone.0117754 (PMC4331085; doi:10.1371/journal.pone.0117754)
Supplement: S1 File — (PDF) [file pone.0117754.s001.pdf]

OPEN ACCESS Freely available online

PLOS | ONE

Correction

Correction: Paraphyly of the Subgenus *Sintonius* (Diptera, Psychodidae, *Sergentomyia*): Status of the Malagasy Species. Creation of a New Subgenus and Description of a New Species

Jérôme Depaquit, Nicole Léger

The type-species is not designated in the published article. The type-species should appear in the Results as: "Type-species of this new subgenus: *Sergentomyia huberti* comb. nov."

Reference

1. Randrianambinintsoa FJ, Léger N, Robert V, Depaquit J (2014) Paraphyly of the Subgenus *Sintonius* (Diptera, Psychodidae, *Sergentomyia*): Status of the Malagasy Species. Creation of a New Subgenus and Description of a New Species. PLoS ONE 9(6): e98065. doi:10.1371/journal.pone.0098065.

Citation: Jérôme Depaquit, Nicole Léger (2014) Correction: Paraphyly of the Subgenus *Sintonius* (Diptera, Psychodidae, *Sergentomyia*): Status of the Malagasy Species. Creation of a New Subgenus and Description of a New Species. PLoS ONE 9(10): e110347. doi:10.1371/journal.pone.0110347

Published October 1, 2014

Copyright: © 2014 Jérôme Depaquit, Nicole Léger. This is an open-access article distributed under the terms of the Creative Commons Attribution License, which permits unrestricted use, distribution, and reproduction in any medium, provided the original author and source are credited.
